# In vitro systems: A new window to the segmentation clock

**DOI:** 10.1111/dgd.12710

**Published:** 2021-03-09

**Authors:** Margarete Diaz‐Cuadros, Olivier Pourquie

**Affiliations:** ^1^ Department of Genetics Harvard Medical School Boston Massachusetts USA; ^2^ Department of Pathology Brigham and Women’s Hospital Boston Massachusetts USA; ^3^ Harvard Stem Cell Institute Boston Massachusetts USA

**Keywords:** paraxial mesoderm, pluripotent stem cells, segmentation clock

## Abstract

Segmental organization of the vertebrate body plan is established by the segmentation clock, a molecular oscillator that controls the periodicity of somite formation. Given the dynamic nature of the segmentation clock, in vivo studies in vertebrate embryos pose technical challenges. As an alternative, simpler models of the segmentation clock based on primary explants and pluripotent stem cells have recently been developed. These ex vivo and in vitro systems enable more quantitative analysis of oscillatory properties and expand the experimental repertoire applicable to the segmentation clock. Crucially, by eliminating the need for model organisms, in vitro models allow us to study the segmentation clock in new species, including our own. The human oscillator was recently recapitulated using induced pluripotent stem cells, providing a window into human development. Certainly, a combination of in vivo and in vitro work holds the most promising potential to unravel the mechanisms behind vertebrate segmentation.

## Graphical abstract

Segmental organization of the vertebrate body plan is established by the segmentation clock, a molecular oscillator that controls the periodicity of somite formation. Given the dynamic nature of the segmentation clock, in vivo studies in vertebrate embryos pose technical challenges. Here, we review the emergence of ex vivo and in vitro models of the segmentation clock as complementary study systems.

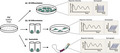

## 1 INTRODUCTION

During vertebrate development, segmentation along the anterior‐posterior body axis is established by the periodic formation of somites. A molecular oscillator known as the segmentation clock is at work in presomitic mesoderm (PSM) cells and underlies the rhythmic nature of somitogenesis (Hubaud & Pourquié, 2014). The segmentation clock is composed of a network of genes whose expression cyclically travels along the PSM in posterior‐to‐anterior wave‐like patterns (Aulehla et al., 2008; Bessho et al., 2003; Palmeirim et al., 1997). In the anterior region of the presomitic mesoderm, the segmentation clock establishes the future somite boundaries by interacting with a determination front positioned by gradients of FGF and Wnt signaling (Aulehla et al., 2003; Dubrulle et al., 2001). Even though more than 20 years have passed since the initial discovery of the segmentation clock (Palmeirim et al., 1997), multiple fundamental questions remain only partially answered. For instance, what is the precise mechanism underlying segmentation clock oscillations? What sets the pace of these oscillations? How are traveling waves generated? How can signaling gradients be reconciled with oscillatory pathway activity? How exactly is the determination front encoded and read by cells? What is the role of phase gradients and phase shifts between oscillators?

Some of these questions have begun to be tackled in more quantitative, precise ways thanks to the development of ex vivo and in vitro models of the segmentation clock. Dynamic information is crucial for the study of the segmentation clock, but somitogenesis‐stage embryos can be difficult to maintain under experimental conditions and live reporter lines require time‐consuming engineering of transgenic animals. Thus, even though embryos represent the only complete model of somitogenesis, the use of explants and pluripotent stem cell (PSC)‐derived models can help usher in technological advances that will accelerate discoveries. In the past decade, we have seen the establishment of the first mouse presomitic mesoderm explant systems, as well as the recapitulation of the segmentation clock using both mouse and human PSCs (Diaz‐Cuadros et al., 2020; Hubaud et al., 2017; Lauschke et al., 2013; Matsuda et al., 2020; Matsumiya et al., 2018). Notably, the human segmentation clock was visualized for the first time thanks to the directed differentiation of PSM cells from induced pluripotent stem cells (iPSCs) (Chu et al., 2019; Diaz‐Cuadros et al., 2020; Matsuda et al., 2020). Here, we review the trajectory, significance, and prospects of modeling vertebrate segmentation in the dish.

## 2 SOMITOGENESIS AND THE SEGMENTATION CLOCK

**FIGURE 1:**
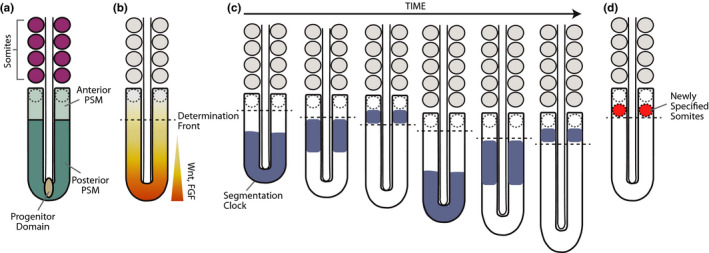
The Segmentation Clock. (a) A posterior progenitor domain (light brown) gives rise to posterior PSM cells (dark teal). In the anterior PSM (light teal), future somites are specified (dotted circles) and subsequently bud off (purple circles). (b) Parallel signaling gradients of Wnt/FGF (yellow‐orange gradient) position the determination front (dotted line) in the anterior PSM. (c) Oscillations of the segmentation clock appear as traveling waves (blue) emanating from the posterior region. As they approach the determination front (dotted line), waves narrow and slow down. (d) The interaction of the segmentation clock with the determination front leads to segment specification through the activation of *Mesp* genes (red)

Like many other bilaterians, the bodies of vertebrate animals are organized into repeating segments of similar structure along the anterior‐posterior (AP) axis. Segmentation is most evident in the periodic arrangement of vertebrae and ribs along the axial skeleton. However, additional structures such as the associated tendons, ligaments and muscles are also segmented (Pourquié, 2011). Even though the total number and shape of segments vary significantly between species, the genetic and morphogenic mechanisms underlying segmentation are highly conserved across vertebrates (Gomez et al., 2008). Segmentation is first established in the paraxial mesoderm through the formation of somites, which are bilaterally symmetric blocks of epithelial tissue that flank the neural tube on both sides (Figure 1a) (Saga & Takeda, 2001). Somite formation takes place rhythmically and sequentially in the anterior‐most part of the unsegmented PSM (Hubaud & Pourquié, 2014). By virtue of their periodic arrangement, somites represent the blueprint for segmentation of the musculoskeletal axis, the vascular system and the peripheral nervous system in vertebrate embryos. Signaling molecules secreted by surrounding tissues drive somites to further differentiate into sub‐compartments that give rise to different lineages, including the dermatome (dermis), myotome (skeletal muscle), sclerotome (bone, cartilage) and syndetome (tendons and ligaments) (Christ & Scaal, 2008). Primary segmentation thus requires the process of somitogenesis to be precisely timed and regulated in order to establish a periodic pattern along the AP body axis.

The process of somite formation takes place with a fixed, species‐specific rhythm known as the somitogenesis period. This period varies significantly between species: 30 min in zebrafish (Schröter et al., 2008), 90 min in chicken (Palmeirim et al., 1997), 2.5 hr in mouse (Tam, 1981) and 5 hr in human (Müller & O'Rahilly, 1986). At the molecular level, the somitogenesis period is controlled by the segmentation clock, which is composed of a network of genes whose expression oscillates in the PSM. Traveling waves of gene expression are initiated rhythmically in the posterior end of the embryo and travel along the PSM in a posterior‐to‐anterior direction (Figure 1c). These waves are kinematic in nature, meaning that they are driven cell‐autonomously by a shift in oscillatory phase between neighbors and not by a traveling signal (Masamizu et al., 2006; Palmeirim et al., 1997). Each pulse of the segmentation clock triggers the specification of a new somite pair by interacting with signaling gradients that position a maturation or determination front in the anterior PSM (Figure 1b,c). Posterior‐to‐anterior gradients of FGF and canonical Wnt/β‐catenin signaling specify the level of this determination front (Figure 1b) (Aulehla et al., 2008; Diez del Corral, 2003; Dubrulle et al., 2001). When a specific phase of the segmentation clock reaches this level, Notch activity drives the expression of boundary markers, such as *Mesp2* and *Ripply2*, that demarcate the future segment (Figure 1d) (Oginuma et al., 2008; Saga et al., 1997). Through this clock and wavefront mechanism, the temporal periodicity of the segmentation clock is translated into the spatial pattern of somites.

The segmentation clock is conserved across vertebrates, but its topology and the identity of cyclic genes is remarkably diverse (Krol et al., 2011). Only components of the Notch signaling pathway oscillate in all vertebrate species studied so far (Hubaud & Pourquié, 2014). The most conserved cyclic genes are members of the hairy and enhancer of split (*Her/Hes*) family of basic helix‐loop‐helix transcription factors (Bessho et al., 2003; Giudicelli et al., 2007; Palmeirim et al., 1997). Additional Notch ligands, effectors and targets such as *Delta*, *Lfng* and *Nrarp* oscillate in some species but not others (Krol et al., 2011). The avian and mammalian segmentation clocks display a more complex topology, as targets of the FGF (e.g., *Dusp* and *Spry* genes) and Wnt (e.g., *Axin2*, *Dkk1*) signaling pathways oscillate in addition to Notch components (Aulehla et al., 2003; Dale et al., 2006). These different oscillating pathways cross‐regulate and entrain each other, resulting in intricate regulatory relationships that have not been fully elucidated (Niwa et al., 2007; Sonnen et al., 2018). Given the complexity of the cyclic gene network, a complete understanding of the segmentation clock requires systems‐level studies that have so far remained unfeasible.

Segmentation clock oscillations are thought to arise from delayed negative feedback loops. The basic premise is that the product of cyclic genes can act as negative feedback inhibitors for their own transcription or for the pathway that controls their expression (Lewis, 2003). Oscillations take place because there is a time delay between the activation of the cyclic gene and the accumulation of sufficient protein to cause feedback inhibition. Consistent with this model, the *Hes/Her* core cyclic genes are transcriptional repressors that inhibit their own promoter (Hirata et al., 2002). Furthermore, several cyclic genes in the Wnt and FGF pathways are negative pathway regulators (Aulehla et al., 2003; Dequéant et al., 2006; Hubaud & Pourquié, 2014). Nevertheless, multiple lines of evidence argue that simple delayed negative feedback loops cannot fully explain the clock mechanism. For instance, Wnt target oscillations continue even in the presence of constitutive Wnt or Notch pathway activity (i.e., non‐phosphorylatable β‐catenin) (Aulehla et al., 2008; Feller et al., 2008). Similarly, Notch target oscillations take place in isolated PSM cells, where the lack of cell‐cell contact prohibits Notch signaling, and even in the context of pharmacological Notch inhibition (Diaz‐Cuadros et al., 2020; Hubaud et al., 2017). Further studies are thus needed to draw a comprehensive picture of the mechanisms driving segmentation clock oscillations and their dynamic properties.

In order to trigger segment specification, the segmentation clock must interact with a regressing determination front specified by FGF and canonical Wnt/β‐catenin gradients. The activity of these two signaling pathways is high in the posterior progenitor domain, which corresponds to the regressing primitive streak at early stages and to the tailbud after the trunk‐to‐tail transition. Pathway ligands such as Fgf8, for instance, are actively transcribed only in the progenitor domain and stop being produced once cells ingress into the PSM territory (Dubrulle & Pourquié, 2004). Over time, the progressive degradation of ligand mRNA and protein results in the formation of the posterior‐to‐anterior FGF gradient that accompanies posterior elongation of the body axis. Similar mechanisms have been proposed to establish the Wnt gradient by spatially restricted Wnt3a transcription (Aulehla et al., 2003). Importantly, FGF and Wnt form a positive feedback loop that reinforces and stabilizes the parallel gradients (Stulberg et al., 2012). Interestingly, Wnt activation downstream of FGF signaling involves additional gradients of glycolytic activity and intracellular pH, thus highlighting the important role of metabolism in patterning the vertebrate body axis (Oginuma et al., 2017, 2020). Originally, the determination front was conceptualized as a simple maturation wave (Cooke & Zeeman, 1976). Subsequently, the determination front was defined as a threshold of the PSM FGF and Wnt signaling gradients (Aulehla et al., 2003; Dubrulle et al., 2001; Saga & Takeda, 2001). Nevertheless, whether such a simple threshold really exists and how cells read out the threshold remains unclear. It has been suggested that cells might instead read the spatial fold change in FGF signaling, or that opposing RA‐FGF gradients might generate a bistability domain that allows coordinated segment specification (Goldbeter et al., 2007; Simsek & Özbudak, 2018).

Vertebrate segmentation thus relies on two distinct but interacting entities: the segmentation clock and the determination front. Despite decades of extensive research, multiple fundamental questions concerning the nature of the clock and wavefront remain unanswered. Most of what we know about vertebrate segmentation has been derived from in vivo studies in zebrafish, chicken, and mouse embryos. Unfortunately, somitogenesis‐stage embryos provide limited PSM material, such that the breadth of experimental techniques directly applicable to embryos remains limited. Additionally, the embryos of some species, such as mouse, can be difficult to culture under experimental conditions. Other species, like the chicken, are not amenable to genetic engineering. Such limitations have motivated researchers to look beyond the embryo for alternative model systems of vertebrate segmentation.

## 3 THE ROAD TO IN VITRO: EXPLANT CULTURES AND PRIMARY CELL‐BASED SYSTEMS

As a first alternative to complement in vivo work, ex vivo explants and primary PSM culture systems were initially developed. In fact, the use of explant cultures is inextricably linked to the study of the segmentation clock as they were involved in its discovery. Oscillations of the segmentation clock were first demonstrated in chicken embryos by performing in situ hybridization for the gene *cHairy1* in caudal explants that were bisected along the midline (Palmeirim et al., 1997). One half was fixed immediately, and the other was allowed to continue developing on filters for a specific amount of time. These experiments demonstrated that *cHairy1* is expressed in a cyclic fashion with a period that matches somitogenesis. Furthermore, explants consisting of the posterior part of mouse embryos, encompassing the tailbud, PSM and several somites, were also employed for the study of somitogenesis early on. Such PSM explant cultures enabled timelapse imaging of the first live segmentation clock reporters (Masamizu et al., 2006). Thus, explant systems have represented an important experimental platform for the segmentation clock ever since the field was first established.

### 3.1 mouse tailbud explants: Monolayer PSM and stably oscillating systems

**FIGURE 2:**
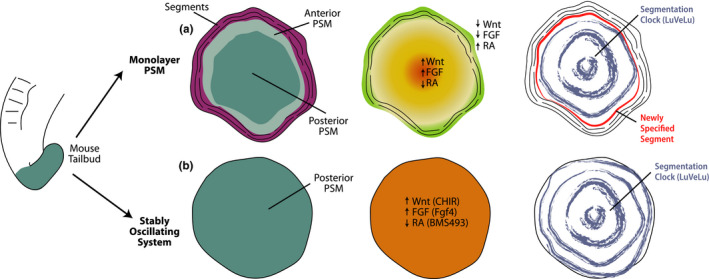
PSM Explant Systems. (a) Monolayer PSM explants recapitulate segmentation along the central‐peripheral axis. Posterior PSM is located centrally (dark teal), surrounded by anterior PSM (light teal) and finally by segmented tissue (purple). Central‐peripheral signaling gradients are also formed (FGF/Wnt‐yellow; RA‐green). Oscillations of the segmentation clock generate concentric waves that travel outwards (blue). Segment specification occurs peripherally in a *Mesp2*‐expressing domain (red). (b) Stably oscillating explants mimic the signaling environment in the posterior region of the embryo to maintain a pure posterior PSM population (dark teal). No signaling gradients are observed in these explants (orange). Oscillations are sustained in the form of concentric waves that travel outwards (blue)

Early explant systems maintained the original tissue topology intact, including the tridimensional organization and developmental sequence. In more recent years, simpler quasi two‐dimensional explant systems have been developed where novel spatiotemporal patterns of segmentation clock oscillations and signaling gradients are established. The first of these quasi‐2D models consisted of tailbud mesoderm explants cultured on fibronectin using a minimal medium without serum supplementation (Lauschke et al., 2013). As explants attached and expanded onto the dish, they formed a flat disk that was termed “monolayer PSM” (Figure 2a). By using mouse embryos of the *Lfng* reporter line *LuVeLu*, oscillations of the segmentation clock could be readily monitored in monolayer PSM explants. The novel topology gave rise to concentric waves of *LuVeLu* expression that traveled outward from the center of the explant (Lauschke et al., 2013). The direction of traveling waves suggested that the posterior‐anterior axis of the embryo had been replaced by a central‐peripheral axis in monolayer PSM explants. Indeed, Wnt and FGF targets were highly expressed in the center, whereas RA synthesis genes were mainly expressed in the periphery (Figure 2a). After a series of oscillations, the explant periphery began to express boundary markers such as *Mesp2* and exhibited morphological segment formation (Figure 2a). As new segments were laid down, the central PSM region progressively shrank and signaling gradients regressed (Lauschke et al., 2013). This novel explant system thus retained an active segmentation clock, determination front and sequential segment formation but displayed an entirely new, circular, and two‐dimensional topology. The flat shape of monolayer PSM explants greatly simplified timelapse imaging of the segmentation clock and enabled quantitative assessment of oscillatory dynamics and phase shifts. Notably, similar explants can be generated from the zebrafish tailbud and display stable oscillations (Webb et al., 2016).

The monolayer PSM system has since given rise to several different adaptations that serve specific and important purposes. For instance, monolayer PSM explants can be combined with microfluidics devices to assess the role of external cues, such as signaling molecules, in regulating the dynamic properties of the segmentation clock. Using microfluidics to provide pulses of pharmacological modulators of the Notch and Wnt signaling pathway, monolayer PSM explants have been entrained to oscillate with an exogenously imposed period and they have been used to demonstrate reciprocate entrainment of the Notch and Wnt oscillators (Sonnen et al., 2018). Furthermore, the phase relationship between cyclic genes has also been successfully manipulated (Sonnen et al., 2018). An additional application of microfluidics will potentially be the imposition of artificial signaling gradients with precisely determined spatiotemporal profiles. Implementation of microfluidics technologies to the study of vertebrate segmentation thus represents a promising development that holds tremendous potential to move the field forward.

A second adaptation of the original monolayer PSM system was the development of stably oscillating posterior PSM explants (Figure 2b). By altering the medium composition to mimic the signaling environment of the posterior PSM, mouse tailbud explants can be maintained in a stable oscillating state for more than 2 days without segment specification (Hubaud et al., 2017). Specifically, this state can be achieved by supplementing the culture medium with Fgf4, the Wnt agonist CHIR99021, the RA inhibitor BMS493, and the BMP inhibitor LDN193189. In contrast to monolayer PSM explants, however, central‐peripheral signaling gradients are not observed and no boundary marker expression or segment formation takes place at the periphery (Figure 2b) (Hubaud et al., 2017). Nevertheless, tailbud explants under these conditions display concentric traveling waves of *LuVeLu* reporter activity with a constant frequency (Figure 2b). This stably oscillating explant system thus represents an ideal experimental model to probe the properties of the segmentation clock and it was used to argue for the excitable properties of the said clock (Hubaud et al., 2017).

### 3.2 culturing dissociated primary PSM cells

Another type of primary cell culture system that completely disrupts the embryonic tissue topology is the culture of dissociated single PSM cells. Culturing individual PSM cells in an isolated state has been a long‐standing goal in the field of vertebrate segmentation, as it would reveal whether the segmentation clock is cell‐autonomous or requires cell‐cell contact for oscillations. In an early study, the chicken PSM was dissected, dissociated, and cultured in suspension (Maroto et al., 2005). Unfortunately, as cyclic gene reporter lines are not available in chicken, cells had to be fixed and in situ hybridization for *Lfng* was performed. This experiment suggested that dissociated cells continue oscillating but lose synchrony. When the first mouse transgenic reporter line (*Hes1‐Luciferase*) was developed, PSM cells were similarly dissociated and cultured on poly‐D‐lysine (Masamizu et al., 2006). However, only few cells could be successfully cultured and displayed unstable pulses of reporter activity. Early attempts at dissociated PSM cultures were thus only partially successful and inconclusive.

Dissociated PSM cultures were optimized in zebrafish by addition of Fgf8 to the culture medium (Webb et al., 2014, 2016). In these experiments, the tailbud was dissected from embryos of the *Looping* zebrafish line, which expresses a Her1‐YFP cyclic gene reporter (Soroldoni et al., 2014). The explants were then trypsinized and cells were plated at low density on fibronectin‐coated dishes (Webb et al., 2016). Initially, single PSM cells only displayed approximately two pulses before quickly dampening. Including Fgf8 in the culture medium to maintain the posterior PSM fate enabled sustained oscillations. In isolated cells, the period of oscillations was significantly longer than in vivo (~75 min vs. ~30 min). Furthermore, oscillations in isolated cells were less precise and persistent than in vivo, often starting and stopping or skipping a cycle. This dissociated zebrafish PSM culture system demonstrated that segmentation clock oscillations are cell‐autonomous, but they require tissue coupling to fine‐tune their period and persistence.

In mouse, the stably oscillating explant system described above also enabled the culture of dissociated cells (Hubaud et al., 2017). These explants were dissociated with Accutase and similarly plated at low density on fibronectin‐coated dishes in media mimicking the posterior PSM signaling environment. Under these conditions, PSM cells adopted a flattened morphology and ceased to express the *LuVeLu* reporter. A critical cell density requirement for oscillations was demonstrated by culturing the cells on fibronectin micropatterns and carefully controlling the number of cells per micropattern (Hubaud et al., 2017). Moreover, a series of experiments elucidated that elevated Hippo/Yap signaling levels in isolated cells induces a quiescent state. Consequently, Yap inhibition restored oscillations in isolated cells. Based on these observations, it was proposed that the segmentation clock is an excitable system where Yap controls the excitability threshold and Notch signaling provides the stimulus. In this case, the combination of a stably oscillating system with single cell culture brought about a breakthrough in our understanding of the underlying nature of the segmentation clock.

### 3.3 dissociation and re‐aggregation

An important application of mouse explant systems has been the dissociation and re‐aggregation of PSM cells to probe their self‐organization capacity. In an interesting study, the entire PSM including both posterior and anterior regions was dissected and dissociated (Tsiairis & Aulehla, 2016). By generating a single cell suspension where all PSM cells were mixed together, positional information was lost and cells were randomized. Dissociated cells were then reaggregated by centrifugation, carefully cut into small pieces, and plated on fibronectin. Despite the randomization, regularly spaced foci appeared within reaggregated cultures. The foci resembled miniature monolayer explants and were termed emergent PSM (Tsiairis & Aulehla, 2016). Each focus displayed target wave patterns, high Wnt activity in the center, and *Mesp2* expression in the periphery. Further experiments demonstrated that there is no special pacemaker cell population and that the oscillation dynamics depend on the phase and frequency of the input cells. Similar dissociation‐aggregation experiments were conducted using the stably oscillating system of mouse PSM explants (Hubaud et al., 2017). In this case, the reaggregated cells also recapitulated the oscillation dynamics of intact explants and quickly synchronized despite including cells from multiple different embryos (Hubaud et al., 2017). Together, these studies highlighted the self‐organization abilities of PSM cells and the central role of Notch‐based synchronization in the emergence of collective oscillations.

## 4 DIRECTED DIFFERENTIATION OF PSM CELLS FROM PLURIPOTENT STEM CELLS

The successful culture of PSM explants indicated that the fate trajectory of PSM cells, the segmentation clock and segment determination could all take place in an in vitro context. This opened the door to the generation of completely in vitro systems based on the differentiation of PSCs towards PSM fate. In fact, the induction of paraxial mesoderm cells from PSCs was desirable not only for the study of vertebrate segmentation, but also as a starting point for skeletal muscle differentiation (Chal & Pourquié, 2017; Pourquié et al., 2018). However, paraxial mesoderm was not as easily derived in vitro as extraembryonic or cardiac mesoderm (Kaufman et al., 2001). Traditional protocols for mesoderm induction relied on BMP and TGFβ activation but resulted in a low yield of paraxial mesoderm cells (Sakurai et al., 2012). It was thus necessary to recapitulate the specific signals that regulate paraxial mesoderm specification in vivo to successfully generate these cells in vitro.

### 4.1 PSM progenitors: The anterior primitive streak

**FIGURE 3:**
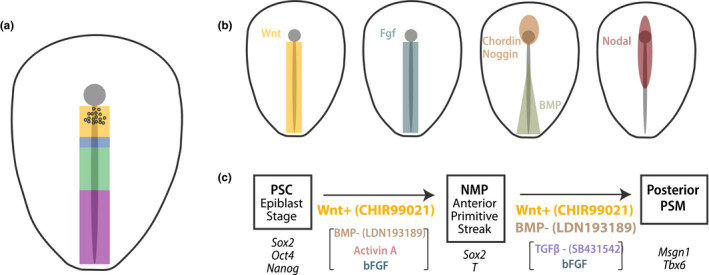
Directed differentiation of PSM cells. (a) Fate‐map of the early primitive streak in chicken embryos. Paraxial mesoderm is derived from the anterior region of the streak. NMPs are shown as circles in the node‐streak border and caudal lateral epiblast (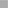, Notochord; 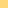, Paraxial Mesoderm; 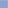, Intermediate Mesoderm; 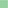, Lateral Plate; 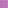, Extraembryonic; 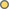, NMPs). (b) Spatial patterns for the Wnt (yellow), FGF (blue), BMP (green/brown), Activin/Nodal/TGFβ (maroon) signaling pathways in the primitive streak. NMPs and paraxial mesoderm precursors experience high Wnt, high FGF, low BMP and moderate TGFβ signaling. (c) Two‐step differentiation protocol for the generation of posterior PSM cells from epiblast‐stage PSCs. Wnt activation is indispensable in the first step, whereas both Wnt activation and BMP inhibition are needed in the second step.

In mouse and chicken embryos, paraxial mesoderm is derived from a population of precursor cells in the anterior region of the primitive streak and its lateral epiblast (Figure 3a). This region contains lineage‐restricted progenitors as well as neuromesodermal progenitors (NMPs) (Guillot et al., 2020; Iimura et al., 2007; Romanos et al., 2020; Tzouanacou et al., 2009). Even though NMPs have only been recently described and remain to be fully characterized, they can be defined as transcriptionally distinct progenitor cells that have the potential to give rise to both paraxial mesoderm and neural tube lineages (Wilson et al., 2009). NMPs are commonly identified by the co‐expression of the mesodermal marker *T/Brachyury* and the neural marker *Sox2* (Henrique et al., 2015 *)*. However, levels of SOX2 and T vary between progenitor cells and can bias the differentiation potential of these cells towards the mesodermal or neural lineage (Kawachi et al., 2020; Romanos et al., 2020). Furthermore, even though NMPs have been described in vivo in zebrafish, chicken, and mouse embryos, the degree of evolutionary conservation of this cell type remains poorly understood (Attardi, 2018; Guillot et al., 2020; Tzouanacou et al., 2009). Nevertheless, both lineage‐committed mesodermal precursor cells and NMPs share a similar signaling environment in the anterior primitive streak (Figure 3a). Understanding this signaling environment, which drives cells towards mesodermal differentiation, was crucial to generate paraxial mesoderm cells in vitro.

The anterior primitive streak is characterized by high levels of FGF and Wnt signaling (Figure 3b). Progenitor cells actively express both FGF (e.g., Fgf4, Fgf8) and Wnt (e.g., Wnt3a) ligands, as well as target genes corresponding to both pathways (Aulehla et al., 2003; Chapman, 2004; Dubrulle & Pourquié, 2004). In fact, the mesodermal marker *T/Brachyury* is a direct Wnt target itself (Arnold et al., 2000). In addition to Wnt and FGF, the BMP inhibitors *Chordin* and *Noggin* are expressed by the node, just anterior to the PSM progenitor domain (Figure 3b) (Streit & Stern, 1999). In contrast, the posterior streak fated to give rise to intermediate, lateral, and extraembryonic mesoderm is characterized by increasing levels of BMP signals such as BMP4 (Figure 3b) (Nostro et al., 2008; Robertson, 2014). Lastly, Activin/Nodal/TGFβ signaling is also active in the anterior primitive streak and is involved in the specification of anterior derivatives (Figure 3b) (Robertson, 2014; Zinski et al., 2018). The signaling environment characterizing the anterior primitive streak thus corresponds to high Wnt, high FGF, low BMP and moderate TGFβ.

Based on the signaling cues gleaned from in vivo studies, anterior primitive streak precursors and NMPs have been efficiently induced from epiblast‐stage PSCs. Invariably, the only indispensable factor required for the generation of T^+^ progenitors is Wnt activation (Figure 3c) (Henrique et al., 2015). This can be achieved by treatment with the GSK3β inhibitor CHIR99021, which leads to β‐catenin stabilization through inhibition of the destruction complex (Chal et al., 2015; Edri et al., 2019; Gouti et al., 2014; Tsakiridis et al., 2014; Turner et al., 2014). Although bFGF is also included in multiple differentiation protocols and can improve efficiency, Wnt activation is sufficient to induce FGF ligand production by the cells themselves and results in robust FGF pathway stimulation (Denham et al., 2015; Diaz‐Cuadros et al., 2020). To anteriorize the resulting primitive streak population, several protocols additionally include Activin A (Craft et al., 2015; Diaz‐Cuadros et al., 2020; Loh et al., 2016; Matsuda et al., 2020; Tsakiridis et al., 2014; Turner et al., 2014; Xi et al., 2017) or BMP inhibitors (Chal et al., 2015, 2016, 2018; Matsuda et al., 2019, 2020). However, much like in the case of FGF, Wnt activation is sufficient to promote Nodal expression by the cells themselves. In most cases, T^+^ progenitor induction from epiblast‐stage mouse and human PSCs is fast (~24–48 hr) and efficient (>80%) (Chal et al., 2015; Diaz‐Cuadros et al., 2020; Gouti et al., 2014; Tsakiridis et al., 2014). NMP cells derived from epiblast‐stage PSCs strongly resemble in vivo NMPs transcriptionally and epigenetically (Diaz‐Cuadros et al., 2020; Edri et al., 2019; Gouti et al., 2014; Metzis, 2018), but it remains unclear whether significant differences might exist between in vitro and in vivo NMPs.

### 4.2 PSM induction

Once PSCs have been converted into mesodermal precursors, these cells can be directed to differentiate into PSM. Again, Wnt activation is the most important factor in this differentiation step (Figure 3c) (Chal et al., 2015, 2016; Henrique et al., 2015; Loh et al., 2016; Pourquié et al., 2018). In vivo, Wnt signaling is required for NMPs to acquire a mesodermal rather than neural fate (Henrique et al., 2015). Mouse mutants for Wnt ligands and targets form ectopic neural tissue at the expense of PSM (Chapman & Papaioannou, 1998; Galceran et al., 1999; Yoshikawa et al., 1997). Thus, maintaining progenitors in Wnt‐inducing medium leads to mesoderm induction (Chal et al., 2015; Loh et al., 2016; Nakajima et al., 2018; Xi et al., 2017). However, at this step, modulators of additional pathways are required to ensure the paraxial identity of induced mesodermal cells, most often defined by expression of *Msgn1*, *Tbx6* and *Dll1*. This is most efficiently achieved by BMP inhibition (Figure 3c) (Chal et al., 2015; Matsuda et al., 2020; Nakajima et al., 2018). Additionally, as TGFβ inhibitors (e.g., follistatin) are expressed in the early PSM (Chapman, 2002), some differentiation protocols block the TGFβ pathway (Chu et al., 2019; Matsuda et al., 2019, 2020; Nakajima et al., 2018). PSM induction only takes one additional day and can be highly efficient, with some protocols reaching upwards of 90% efficiency (Chal et al., 2018). Given that the signals required for anterior primitive streak induction and PSM specification are remarkably similar, several differentiation protocols have adopted a one‐step approach where the same factors are used for two consecutive days to achieve PSM generation from PSCs (Chal et al., 2015, 2018; Chal & Pourquié, 2017; Matsuda et al., 2019; Nakajima et al., 2018; Xi et al., 2017). Thus, despite the early difficulties in paraxial mesoderm differentiation, PSM cells can now be rapidly and efficiently generated from epiblast‐stage PSCs.

## 5 IN VITRO MODELS OF THE SEGMENTATION CLOCK

### 5.1 segmentation clock organoids and monolayer cultures

**FIGURE 4:**
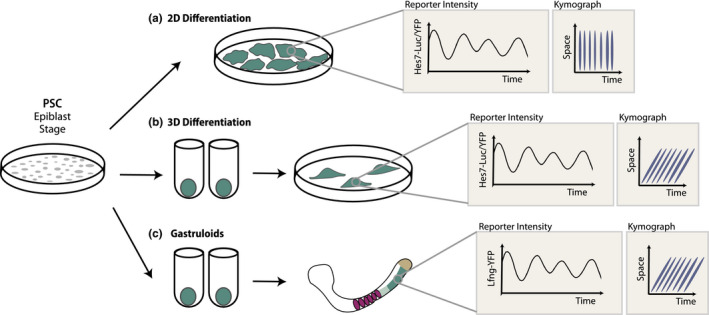
In vitro models of the segmentation clock. (a) Differentiation of PSCs in monolayer culture leads to synchronously oscillating PSM cells. No wave patterns are observed, as indicated by the vertical orientation of lines in the kymograph. (b) Performing the initial steps of PSM differentiation in 3D aggregates and subsequently allowing these aggregates to spread on culture dishes leads to wave‐like patterns of segmentation clock oscillations. Traveling waves can be seen as lines with a slanted slope in the kymograph. (c) Gastruloids can recapitulate traveling waves of the segmentation clock coupled with morphological somite formation when embedded in low‐percentage Matrigel. Traveling waves can be seen as lines with a slanted slope in the kymograph. Light brown: progenitor domain, dark teal: posterior PSM; light teal: anterior PSM; purple: somites

The establishment of directed differentiation methods for PSM induction from PSCs provided a foundation for the modeling of higher‐order and more complex biological processes in vitro. The first of such studies was conducted using mouse embryonic stem cells (mESCs) (Matsumiya et al., 2018). These cells were seeded in non‐adherent dishes to form 3D aggregates and then pre‐differentiated to an epiblast‐like state by treatment with BMP4. The aggregates were then transferred to gelatin‐ or fibronectin‐coated dishes and allowed to attach. At this step, the medium was changed to a combination of CHIR99021 and LDN193189 for PSM induction. After two days, the aggregates, termed induced PSM or iPSM, displayed oscillatory *Hes7* activity as revealed by a luciferase‐based reporter (Matsumiya et al., 2018). These oscillations formed traveling waves emanating from the center of each iPSM (Figure 4b). Importantly, oscillations took place with similar period as in vivo, thus indicating that segmentation clock dynamics are conserved in vitro. The central region of iPSMs also displayed the highest levels of FGF signaling, suggesting that signaling gradients were also recapitulated in this system. In the periphery, *Mesp2* expression could be observed and morphological segment formation took place when iPSMs were replated on fibronectin. These characteristics made iPSM strongly resemble the topology of monolayer PSM explants (Lauschke et al., 2013). Thus, the iPSM system represented the first completely in vitro model of the segmentation clock and ushered in a new era in the study of vertebrate segmentation.

Recapitulating the segmentation clock from PSCs offered multiple advantages over in vivo work. First, in vitro systems eliminate the need to breed animals and microdissect PSM tissue from embryos, which is labor intensive, time‐consuming and yields a small amount of biological material. Second, PSCs can be easily and quickly genetically modified using CRISPR/Cas9 to generate reporter lines, knock‐out lines or specific point mutations without the need to establish and maintain transgenic animals. Double or triple mutant/reporter lines can be generated in a straightforward way without complicated genetic crosses. In addition, different cell lines can be mixed in precise ratios within a culture dish to create mosaics, which are very difficult to achieve in vivo in vertebrate embryos. Furthermore, highly reproducible in vitro systems are amenable to high‐throughput screening either with chemical libraries or RNAi/CRISPR libraries (Matsumiya et al., 2018). In vitro models are also easily combined with optogenetics and microfluidics approaches. Lastly, PSCs are not limited to model organisms as they can be derived from the somatic cells of several different species. This expands our ability to study the segmentation clock across vertebrate species and elucidate the mechanisms controlling species‐specific oscillation periods (Matsuda et al., 2019).

Immediately following this initial success, simpler, two‐dimensional models of the mouse segmentation clock have been achieved (Figure 4a) (Diaz‐Cuadros et al., 2020; Matsuda et al., 2019, 2020). For instance, a one‐step differentiation protocol based on Wnt activation and BMP inhibition yields a high percentage (~70%) of PSM cells in monolayer culture (Chal et al., 2015, 2018). These cells undergo oscillations of the segmentation clock that can be monitored by a *Hes7‐Achilles(YFP)* knock‐in reporter (Diaz‐Cuadros et al., 2020). Similarly, a combination of FGF and Wnt activation with BMP and TGFβ inhibition generates synchronously oscillating PSM cells, as revealed by a *Hes7‐Luciferase* transgene (Matsuda et al., 2019). Whereas fluorescent reporter proteins provide cellular resolution, luciferase reporters display much higher intensity. Both of these studies used mouse embryonic stem cells pre‐differentiated to an epiblast‐like stage as the starting material, but mouse epiblast stem cells (EpiSCs) can also give rise to populations of synchronously oscillating PSM cells when subjected to similar differentiation protocols (Matsuda et al., 2020). In these 2D systems, segment specification takes place simultaneously across the culture once oscillations of the segmentation clock have arrested (Diaz‐Cuadros et al., 2020; Matsuda et al., 2020). This means that each culture is not subdivided into regions of oscillating and determined cells, as in the case in the original iPSM (Matsumiya et al., 2018), but rather pure populations of oscillating PSM cells are obtained. In this sense, 2D differentiation systems resemble stably oscillating mouse explants (Hubaud et al., 2017), with the added benefit of an unlimited supply.

Despite their simplicity, 2D models provide multiple technical advantages. Most prominently, monolayer cultures can be produced in much larger quantities than explants or 3D organoids, such that 2D models are amenable to high‐throughput approaches that require large numbers of cells such as metabolomics, ribosome profiling, and bulk RNA‐seq, among others (Matsuda et al., 2020). Moreover, oscillations between different cultures or plates can be synchronized simply by triggering oscillations simultaneously with a medium change (Diaz‐Cuadros et al., 2020; Matsuda et al., 2020). Using this simple trick, high‐throughput approaches can be deployed in samples collected over a time‐series to reconstruct dynamic processes. For example, a comprehensive list of oscillating genes in the mouse segmentation clock was reconstructed by collecting RNA‐seq samples every 30 min from EpiSC‐derived PSM cultures (Matsuda et al., 2020). 2D models of the segmentation clock thus open the door to a host of new experimental approaches to study vertebrate segmentation.

### 5.2 Gastruloids

The segmentation clock has also been recapitulated in vitro within the context of more complex models of post‐occipital embryonic development. Gastruloids are elongated 3D cell aggregates derived from PSCs that contain derivatives of all three germ layers and form dorso‐ventral and anterior‐posterior axes (Beccari et al., 2018; Brink et al., 2014; Turner et al., 2017). A progenitor domain containing NMP‐like cells is located in the posterior region of gastruloids, which elongate along the A‐P axis as they grow. Despite their astonishing complexity, the protocol for gastruloid generation from PSCs is remarkably simple. 3D aggregates are exposed to a pulse of the Wnt agonist CHIR99021 and then allowed to develop without additional exogenous signals (Beccari et al., 2018; Brink et al., 2014; Turner et al., 2017; Veenvliet et al., 2020). Gastruloids contain paraxial mesoderm cells and oscillations of the segmentation clock have been observed using *Lfng‐Venus* mouse ESCs (Brink et al., 2020). These oscillations appear as traveling waves emanating from the posterior progenitor region (Figure 4c). A determination front is located at the anterior boundary of the oscillatory domain and regresses as the gastruloid elongates (Brink et al., 2020). Gastruloids thus faithfully recapitulate the segmentation clock and determination front. Notably, only gastruloids can mimic the anterior‐posterior elongated topology of the embryo, which is commonly replaced by a central‐peripheral axis in other in vitro systems (Figure 4c).

In conventional gastruloids, morphogenetic events such as somite formation do not take place (Beccari et al., 2018). However, recent studies have revealed that embedding gastruloids in low‐percentage Matrigel can induce morphogenesis (Brink et al., 2020; Veenvliet et al., 2020). Under such conditions, one study reported the sequential formation of morphological somites with proper anterior‐posterior polarity (i.e., *Uncx4.1* and *Tbx18* domains) (Brink et al., 2020). Similarly, a second study described the generation of trunk‐like‐structures composed of a neural tube with adjacent paraxial mesoderm cells that also segmented into morphological somites (Veenvliet et al., 2020). The exact role of Matrigel in promoting such complex morphogenesis in gastruloids has not been elucidated, but these conditions certainly result in the closest approximation to an embryonic axis that has been created in vitro. To date, the segmentation clock and somite formation have only been reported in mouse ESC‐derived gastruloids. However, human iPSCs have been recently used to create the first human gastruloids and characterization of their segmental program is highly anticipated (Moris et al., 2020).

### 5.3 modeling the human segmentation clock

Given the success in recapitulating the mouse segmentation clock in vitro, researches raced to model the human segmentation clock using human iPSCs. In vitro models of human development are particularly important due to the inaccessibility of early human embryos and the ethical considerations surrounding them. This is especially true for developmental processes that take place shortly after implantation, such as somitogenesis. Before in vitro systems were developed, very little was known about the human segmentation clock. The somitogenesis period for human embryos was estimated at 4–8 hr based on rare fixed samples (Müller & O'Rahilly, 1986). In addition, genetic studies of human patients had revealed that mutations in segmentation clock genes, such as *Hes7*, *Lfng* and *Dll3*, result in congenital scoliosis and other segmentation defects of the vertebrae (Gucev et al., 2010; Sparrow et al., 2013; Turnpenny et al., 2007, 2008). However, the dynamic nature of the segmentation clock means that we will never observe it directly in human embryos, as this would require the generation of transgenic reporter lines. Thus, in vitro systems represent the only viable option for the study of the human segmentation clock.

Using human iPSCs as a starting point, researchers have now recapitulated the human segmentation in both two‐ and three‐dimensional cultures. Using 2D differentiation protocols that induce a remarkably high percentage of PSM cells from human iPSCs (>90%), the human segmentation clock has been visualized in cells carrying HES7‐Achilles(YFP) and HES7‐Luciferase reporters (Chu et al., 2019; Diaz‐Cuadros et al., 2020; Matsuda et al., 2019, 2020). As is the case for mouse ESC‐derived PSM cells in 2D, synchronous oscillations that are triggered by medium change can be observed across the culture. Traveling waves of human segmentation clock oscillations have also been observed by differentiating cells in 3D aggregates and subsequently allowing them to attach and spread on a dish (Matsuda et al., 2020). In agreement with estimates from human embryos, the human segmentation clock period was measured to be approximately 5 hr in both 2D and 3D cultures (Chu et al., 2019; Diaz‐Cuadros et al., 2020; Matsuda et al., 2019, 2020). These systems thus provided us with the first glimpse of the human segmentation clock.

We have learned much about human segmentation by studying these in vitro models. For example, Notch signaling synchronizes oscillations between neighboring human PSM cells, as is the case in vivo in mouse embryos (Diaz‐Cuadros et al., 2020). Furthermore, the human segmentation clock is also sensitive to levels of Yap signaling and appears to exhibit excitable properties similar to the mouse oscillator (Diaz‐Cuadros et al., 2020). An extensive list of human cyclic genes was constructed through a time‐series of bulk RNA sequencing and revealed the topology of the human segmentation clock (Matsuda et al., 2020). As expected, components of the Notch, Wnt and FGF pathways were observed to oscillate. However, the specific oscillatory genes within each pathway differed from those in mouse and multiple human‐specific cyclic genes were found. These insights would not have been possible without the development of in vitro systems.

In vitro models can recapitulate not only the human segmentation clock but also the determination front. Combining a segmentation clock reporter (*HES7‐Achilles*) with a segment specification reporter (*MESP2‐mCherry*), the transition from oscillatory to segmental fate was directly visualized in human PSM cells (Diaz‐Cuadros et al., 2020). The timing of segment determination (i.e., MESP2 activation) depended on the levels of FGF and Wnt signaling and could be readily manipulated by inhibitors of these pathways. This suggested that human PSM cells in vitro experienced a temporal determination front. Indeed, Wnt and FGF levels were high in the early phase of differentiation and were later autonomously downregulated, reaching their lowest levels at the time of segmental fate specification (Diaz‐Cuadros et al., 2020). These experiments confirmed that the human determination front is also regulated by the Wnt and FGF signaling pathways. In addition, these in vitro experiments demonstrated that simple 2D systems still recapitulate signaling gradients and the determination front, despite a complete lack of spatial patterning. In fact, differentiating human iPSCs also recapitulate the metabolic transitions that characterize PSM differentiation in mouse and chicken embryos (Oginuma et al., 2017, 2020). As is the case in vivo, signaling gradients are paralleled by decreasing gradients of glycolysis and intracellular pH in vitro (Oginuma et al., 2017, 2020). Moreover, pharmacological inhibition of FGF signaling in human iPSC‐derived PSM cells revealed an unexpected role for FGF in controlling the phase and period of segmentation clock oscillations (Diaz‐Cuadros et al., 2020). Given that the classical clock and wavefront model considered the role of FGF to be limited to the positioning of the determination front, this new finding led to a revision of the current model.

In vitro recapitulation of the human segmentation clock has also allowed us to model human diseases in the dish. In particular, human iPSCs can be used to study genetic diseases that give rise to segmentation defects of the vertebrae (Chu et al., 2019; Matsuda et al., 2020). Not only can phenotype‐causing mutations be genetically engineered into human iPSCs, but patient‐derived iPSC lines can also be established. Such genetically modified or patient‐derived lines can then be differentiated into PSM and potential defects in the segmentation clock can be determined. For example, an iPSC line was established from cells donated by a patient with segmentation defects of the vertebrae and a homozygous mutation in the cyclic gene *DLL3* was found (Matsuda et al., 2020). When differentiated to PSM, the patient‐derived cells could sustain oscillations in 2D but failed to synchronize properly or generate traveling waves in 3D. Correcting the putative disease‐causing mutation in one allele of *DLL3* resulted in the reversal of this phenotype (Matsuda et al., 2020). Through these in vitro experiments, the identity of the disease‐associated variant and its and mechanism of action were elucidated. In vitro systems can thus provide much needed insight into the mechanisms leading to congenital segmentation defects.

## 6 CONCLUSIONS

The development of explant‐based and in vitro models of the segmentation clock have made possible several important discoveries. For example, reciprocal entrainment between Notch and Wnt oscillators was demonstrated using monolayer PSM explants (Sonnen et al., 2018). Stably oscillating explants revealed that the segmentation clock works as an excitable system regulated by Notch and Yap signaling (Hubaud et al., 2017). A role for FGF signaling in regulating the dynamic properties of the segmentation clock was uncovered by using pure PSM populations derived from explants and PSCs (Diaz‐Cuadros et al., 2020). Furthermore, a series of experiments with mouse PSM explants demonstrated that no special pacemaker population exists within the tailbud (Hubaud et al., 2017; Tsiairis & Aulehla, 2016). These conceptual advances would not have been possible without the precise quantification of oscillatory dynamics (phase, period, amplitude, persistence) that are only achievable under controlled in vitro culture conditions.

Furthermore, primary and in vitro derived PSM cells have been cultured as dissociated single cells to establish the cell‐autonomy of segmentation clock oscillations. This type of experiment had been attempted multiple times in the past, but only the establishment of proper culture conditions allowed robust conclusions to be drawn. We now know that oscillations are an autonomous property of PSM cells, but that the period and precision of oscillations depend also on cell‐cell contact (Webb et al., 2016). In addition, PSM cells can stop oscillating while retaining their posterior PSM identity when the excitability threshold set by Yap signaling becomes elevated (Hubaud et al., 2017). The long‐standing question of whether oscillations represent an emergent property at the population level or whether PSM cells oscillate intrinsically could thus only be resolved after we learned how to maintain PSM cells in vitro.

Importantly, the human segmentation clock was directly observed for the first time thanks to in vitro differentiation systems and can now be used to model human diseases (Chu et al., 2019; Diaz‐Cuadros et al., 2020; Matsuda et al., 2019, 2020). This represents an exciting achievement in human developmental biology as it provides insight into an aspect of human development that would otherwise remain obscured. More complex and complete models of human development will take the form of gastruloids, which will potentially exhibit not only segmentation clock oscillations and segment specification, but also A‐P patterning, elongation and morphological somite formation.

Ex vivo and in vitro models of the segmentation clock will open many doors for new lines of experimentation. However, researchers will always need to go back to the embryo to validate and confirm their in vitro findings. After all, embryos represent the only comprehensive model of development. We will learn not only from the aspects of embryonic development that we can recapitulate in the dish, but also from those aspects that we cannot. Armed with simplicity and reproducibility, in vitro models will surely become a regular part of the developmental biologist's toolbox in the coming years.
